# The value of functional substrate mapping in ventricular tachycardia ablation

**DOI:** 10.1016/j.hroo.2022.10.013

**Published:** 2022-11-02

**Authors:** Konstantinos Vlachos, Konstantinos P. Letsas, Neil T. Srinivasan, Antonio Frontera, Michael Efremidis, Stelios Dragasis, Claire A. Martin, Ruaridh Martin, Takashi Nakashima, George Bazoukis, Takeshi Kitamura, Panagiotis Mililis, Athanasios Saplaouras, Stamatios Georgopoulos, Stamatios Sofoulis, Ourania Kariki, Stavroula Koskina, Masateru Takigawa, Frédéric Sacher, Pierre Jais, Pasquale Santangeli

**Affiliations:** ∗ Cardiac Pacing and Electrophysiology Department, Hôpital Cardiologique du Haut Lévêque, Pessac, France; † Electrophysiology Department, Onassis Cardiac Surgery Center, Athens, Greece; ‡ INSERM U1045, Institut hostpialo-universitaire–L’institut de rythmologie et modélisation cardiaque, Centre Hospitalier Universitaire de Bordeaux, Université de Bordeaux, Pessac, France; § Department of Cardiac Electrophysiology, Essex Cardiothoracic Centre, Basildon, United Kingdom; || Royal Papworth Hospital NHS Foundation Trust, Cambridge, United Kingdom; ¶ Department of Basic and Clinical Sciences, University of Nicosia Medical School, Nicosia, Cyprus; # Department of Cardiology, Larnaca General Hospital, Larnaca, Cyprus; ∗∗ Laboratory of Cardiac Electrophysiology, General Hospital of Athens Evangelismos, Athens, Greece; †† Cardiovascular Division, Hospital of the University of Pennsylvania, Philadelphia, Pennsylvania

**Keywords:** Ventricular arrhythmias, Catheter ablation, Functional substrate mapping, Ventricular tachycardia, Multielectrode mapping

## Abstract

In the setting of structural heart disease, ventricular tachycardia (VT) is typically associated with a re-entrant mechanism. In patients with hemodynamically tolerated VTs, activation and entrainment mapping remain the gold standard for the identification of the critical parts of the circuit. However, this is rarely accomplished, as most VTs are not hemodynamically tolerated to permit mapping during tachycardia. Other limitations include noninducibility of arrhythmia or nonsustained VT. This has led to the development of substrate mapping techniques during sinus rhythm, eliminating the need for prolonged periods of mapping during tachycardia. Recurrence rates following VT ablation are high; therefore, new mapping techniques for substrate characterization are required. Advances in catheter technology and especially multielectrode mapping of abnormal electrograms has increased the ability to identify the mechanism of scar-related VT. Several substrate-guided approaches have been developed to overcome this, including scar homogenization and late potential mapping. Dynamic substrate changes are mainly identified within regions of myocardial scar and can be identified as local abnormal ventricular activities. Furthermore, mapping strategies incorporating ventricular extrastimulation, including from different directions and coupling intervals, have been shown to increase the accuracy of substrate mapping. The implementation of extrastimulus substrate mapping and automated annotation require less extensive ablation and would make VT ablation procedures less cumbersome and accessible to more patients.

**Key Findings**

▪ In patients with hemodynamically tolerated ventricular tachycardia, activation and entrainment mapping remain the gold standard for the identification of the critical parts of the circuits.
▪ The high incidence of non–hemodynamically tolerated ventricular tachycardia has led to the development of substrate mapping techniques during sinus rhythm.
▪ Multielectrode mapping using small and closely spaced electrodes allows us to delineate late potentials and local abnormal ventricular activities in low-voltage regions related with ventricular tachycardia re-entry.
▪ Mapping strategies incorporating ventricular extrastimulation have been shown to increase the accuracy of substrate mapping for identifying ventricular tachycardia isthmus sites.

## Introduction

Ventricular tachycardia (VT) in the context of structural heart disease–related VT (ischemic or nonischemic etiology) utilizes re-entrant mechanisms that are dependent on the presence of unidirectional block and wavefront re-entry.^1^ Catheter ablation has become a key component in the management of recurrent post-–myocardial infarction VT. In patients with hemodynamically tolerated VT, activation and entrainment mapping of VT remains the gold standard for the identification of the critical parts of the circuits and a successful catheter ablation outcome.^2^ However, activation mapping remains a challenge due to poorly tolerated VT, VT noninducibility, or the presence of an intramural substrate; the presence of an epicardial substrate in cases in which epicardial access is not possible and 3-dimensional circuits that use all planes of the myocardium and cannot be mapped as a single planar wavefront.^3^

These have led to the development of substrate mapping techniques that are largely conducted during sinus rhythm, eliminating the need for prolonged periods of mapping during tachycardia.^1^ Techniques combining substrate-based ablation with activation and entrainment mapping have been shown to be more effective in preventing VT recurrence than targeted ablation of the clinical VT alone.^4^ Substrate ablation strategies that have been developed include scar homogenization,^5^ scar dechanneling, targeting zones of isochronal crowding, and elimination of local abnormal ventricular activities (LAVAs) or late potentials (LPs), defined as isolated high-frequency local electrograms (EGMs) after the offset of the terminal portion of the QRS complex (Table 1).^6^, ^7^, ^8^ However, outcomes of all these methods are similar, with procedural success as low as 47%^3^^,^^9^^,^^10^^,^^11^ and incidence of procedural complications of about 5% to 10%.^12^ Techniques that target all abnormal potentials or regions of scar border zone (SBZ) include ablation of large areas of myocardium either endocardially or epicardially.^13^^,^^14^

**Table 1:** Various electrode configurations, sizes, and relative spacing with commonly used catheters: Advantages and disadvantages

Model	Manufacturer	Electrodes	Tip electrode size (mm)	Ring electrode size (mm)	Spacing (edge-to edge) (mm)	Spacing recorded (center to center) (mm)	Advantages and disadvantages
ThermoCool ST	Biosense Webster	4	3.5	1	1–6–2	3.25	Inability to differentiate near-field from far-field signals in scar and low-voltage regions
ThermoCool/SF	Biosense Webster	4	3.5	1	2–5–2	4.25	Inability to differentiate near-field from far-field signals in scar and low-voltage regions
FlexAbility	Abbott	4	4	1	1–4–1	3.50	Inability to differentiate near-field from far-field signals in scar and low-voltage regions
TactiCath	Abbott	4	3.5	1	2–5–2	4.25	Inability to differentiate near-field from far-field signals in scar and low-voltage regions
MiFi	Boston Scientific	4	4.5	1	1.5	2.50	Microelectrode cofiguratiom: radial on the distal tip
QDOT	Biosense Webster	4	3.5	0.33	1.5	1.5	Microelectrode cofiguratiom: circumferential on the distal tip
PENTARAY	Biosense Webster	20	—	1	2–6–2		(1) Higher mapping density and better substrate definition; (2) higher detection of LAVAs and voltage channels; (3) higher accuracy in identifying and delineating near-field component (LAVAs) and differentiating from far-field signals
IntellaMap Orion (RHYTHMIA mapping system)	Boston Scientific	64	—	0.9 × 0.45	1.6	2.50	Lumipoint algorithm signal using information across the whole mapping window, rather than only the peak signal; these features may enhance human interpretation of the electrogram signals during a case, particularly in which the circuit lies in partial scar with low-amplitude near-field signals
HD Grid (Abbott, Ensite-X)	Abbott	16	2	1	3	3.00 (along, across), 4.23 (diagonal)	HD-wave solution, omnipolar mapping technology

LAVA = local abnormal ventricular activity.

A key element in facilitating VT is the presence of dynamic changes within the substrate that may not be evident during substrate mapping in sinus rhythm. These dynamic changes may play a critical role in the tachycardia mechanism when conduction velocity slows and tissue refractory periods lengthen. Dynamic substrate changes lie mainly within regions of myocardial scar or the SBZ and can be identified as functional delay in electrograms by introducing timed extrastimuli.^15^^,^^16^ These functional substrate mapping techniques are now being increasingly utilized in clinical practice. In this review, we explore key concepts of functional mapping techniques and explore future directions.

## Mapping systems, tools, and challenges

### Multielectrode mapping technology

The fundamental hypothesis of substrate mapping for scar-mediated VT is that surrogates of the isthmus can be identified. These surrogates include electrocardiographic indications for electric discontinuity such as fractionation, split potentials, LPs, and long potentials, also evident as sites displaying activation slowing.^7^ Advances in mapping catheter technology, especially a decrease in electrode size and spacing, have contributed to better arrhythmia substrate characterization.^17^ Multielectrode mapping of abnormal EGMs has increased the sensitivity in identifying scar-related areas.^18^ Small and closely spaced electrodes allow identification of distinct diastolic activity that may not be seen with standard linear catheters. Moreover, automatic detection of abnormal EGMs may help overcome subjective judgment and accelerate the revision of ultra-high-density 3-dimensional maps with many acquired points.

Myocardial scar definition is a key step of any substrate-based ablation approach.^19^ Three voltage cutoffs are typically used to define the tissue characteristics as normal myocardium, defined as a voltage >1.5 mV; dense scar or low-voltage myocardium, defined by a voltage <0.5 mV; and SBZ or intermediate-voltage myocardium, with a voltage between 0.5 mV and 1.5 mV.^5^^,^^20^ These definitions were made using standard ablation and mapping catheters with 4-mm tip electrodes, 1-mm ring electrodes, and 2-mm interelectrode spacing (Table 1).^5^ However, detected EGMs are strictly dependent on electrode size. Multipolar catheters can improve our accuracy in delineating SBZ and low-voltage areas, as low as 0.02 mV, critical for the maintenance of the VT circuit. The importance of SBZ was shown in initial studies in open-heart models demonstrating that ischemic VT originates in the area surrounding the dense scar, while the surgical removal of this tissue could cure 70% to 80% of arrhythmias.^21^ Recently, studies have partially confirmed this observation and have also highlighted that a significant proportion of re-entrant circuit isthmuses exist within the dense scar.^22^ Martin and colleagues^23^ demonstrated that VT circuits were observed to pass through areas of low bipolar voltage, often <0.2 mV. Indeed, the mid-isthmus median near-field signal amplitude was <0.2 mV in sinus or paced rhythm in all cases in which a sinus or paced map was recorded before ablation. This indicates that although VT is commonly seen to exit from the SBZ, low-voltage scar regions are likely to harbor critical parts of the VT circuit in mappable VT. This finding has been confirmed in a further study by our group, showing that isthmuses often lie in regions of dense scar, which, therefore, is composed of not only dead myocardium, but also viable strands of myocardial tissue.^24^ These findings question the voltage criteria and thresholds regarding viable tissue of previous studies using a conventional ablation catheter and create the need for randomized studies to revisit the voltage criteria of dense scar in the era of high-density mapping.

High-density, multielectrode catheters can also enhance the possibility of detailed activation mapping of even poorly tolerated VTs because a complete map is faster to obtain. Recently, it has been shown that there is higher freedom from VT recurrence in patients with a fully mappable diastolic pathway recording after an ablative procedure.^3^

The use of high-resolution mapping technologies may have a role in improving clinical outcomes.^25^ Recently, Wolf and colleagues^25^ reported that substrate modification targeting LAVAs for post–myocardial infarction VT resulted in a substantial reduction of VT storm and implantable cardioverter-defibrillator shocks and up to 49% of patients free from ventricular arrhythmias (VAs) at 5 years after a single procedure. Complete LAVA elimination, multielectrode mapping, and real-time integration were associated with improved VA-free survival.^25^ Improved mapping by implementing modern technologies such as multielectrode mapping catheters and integration of high-definition imaging of scar and anatomy improved both short- and long-term outcomes, with freedom of VA recurrence in 86% of patients at 1 year and 65% at 4-year follow-up.^25^ Berte and colleagues^26^ elegantly demonstrated that the a multipolar mapping catheter is superior for LAVA detection when compared with a conventional ablation catheter. The higher LAVA detection rate is attributable due to not only a higher mapping density, but also a higher bipolar LAVA amplitude, both within the scar and in the border zone. Therefore, even in the presence of a comparable mapping density, mapping with an ablation catheter is predicted to miss some of the LAVA signals due to a lower amplitude. Intuitively, more accurate identification of LAVAs would be predicted to improve outcome.^26^

The advantages of multipolar catheters (PENTARAY [Biosense Webster, Irvine, CA]/OCTARAY [Biosense Webster]/HD Grid [Abbott, Minneapolis, MN]/Orion [Boston Scientific, Marlborough, MA]) are (1) higher mapping density and better substrate definition; (2) higher detection of LAVAs and voltage channels; and (3) higher accuracy in identifying and delineating near-field component (LAVAs) and differentiating from far-field signals.^24^^,^^26^, ^27^, ^28^ Often, VTs in patients with a structural heart disease are hemodynamically not tolerated and show multiple morphologies during the procedure, preventing the achievement of an activation map. The use of multielectrodes allows the mapping of the VTs to be performed in a reasonable time frame. The capability of demonstrating an effective abolition during sinus rhythm of potentials with a proven participation into the re-entry mechanism (recorded during diastole in VT) opens new perspectives on the assessment of substrate modification, overcoming the limitations of a purely timing-based strategy of LP or LAVA elimination.^28^ The multipolar grid mapping catheter is a suitable tool to complete the VT activation map before hemodynamic deterioration. Specific algorithms such as the Lumipoint algorithm automatically highlight areas with EGMs having specific characteristics or timings. This can identify late and fractionated potentials and regions that exhibit discontinuous activation, as well as the isthmus of a VT circuit. These features may enhance human interpretation of the EGM signals during a case, particularly in which the circuit lies in partial scar with low-amplitude near-field signals, and potentially allow a more targeted ablation strategy.^27^ The Lumipoint algorithm has the advantage of using signal information across the whole mapping window, rather than using only the peak signal. In highlighting regions with activity in the post-QRS phase of a substrate map, an LP map can be automatically generated, which corresponds well with manual annotation. Furthermore, specific areas showing late and fractionated signals can be automatically annotated using the complex activation search feature. This has the potential to allow more rapid mapping, as points do not have to be manually annotated, and reduces the risk of missing some regions.^27^ The Lumipoint algorithm may also identify the potential isthmus of a VT circuit by highlighting the region of the map corresponding to a minimum of activating tissue. VT circuits are often found to occur in regions of partial or even dense scar. In these cases, in which the circuit has low-amplitude near-.field signals, the far-field signal is often automatically annotated, which may mask the true isthmus. This algorithm may aid delineation of the isthmus to guide ablation without manual reannotation.^27^ New mapping tools, with better definitions, or computational insights (mathematical models) may define such features in the near future.

#### Ripple mapping

Ripple mapping is a mapping tool that shows both voltage and propagation channels into the scar in a single dynamic display. This technology has the advantage of displaying complex or continuous fractionated local EGMs as time-dependent propagation, rather than choosing a single annotation for timing. Jamil-Copley and colleagues^29^ were the first to show the potential utility of this technique. Recently, Katritsis and colleagues^30^ used Ripple mapping in scar dechanneling to identify channels of activation within the scar. They analyzed clusters of ripple bars activating simultaneously in SBZ regions and identified LAVAs as a potential target for ablation. Ablation at channel entrance sites eliminated the scar-related LAVAs without direct ablation.^31^ These findings provide further evidence of interconnected channels within postinfarct scar as an effective target for substrate modification.^30^

### The value of EGM morphology to identify the active circuit

Fractionated EGMs are recorded in regions where infarct healing causes wide separation of individual myocardial fibers while distorting their orientation. These anatomic changes likely cause slow and inhomogeneous activation.^32^^,^^33^ Wall thickness in post–myocardial infarction scar is heterogeneous, with channels of relatively preserved thickness bordered by thinner scar. Takigawa and colleagues^34^ recently demonstrated that VT isthmuses were always found in 3-dimensionally reconstructed computed tomography (CT) channels, and half of CT channels hosted VT isthmuses; longer and thinner (but >1 mm) CT channels were significantly associated with VT isthmuses. In human VT, mid-isthmus EGM deflections are typically low in voltage.^35^ Based on the formulation of the extracellular signal, the thinness of the viable myocardium is directly responsible for the diminished amplitude observed in the EGM deflections because of the decreased volume of activating myocytes.^35^^,^^36^ Central or mid-isthmus EGMs also tend to be relatively short in duration,^36^ indicative of a faster activation wavefront. Although the wavefront is slowed overall in the infarct border zone and in the circuit, the isthmus long axis tends to orient approximately in parallel to muscle fibers, in which direction the wavefront conduction velocity is fastest, which would account for the faster speed observed during VT.^35^ Anter and colleagues^17^ reported that conduction velocities are slowest at the inward curvature into the isthmus entrance (0.28 ± 0.2 m/s), slightly faster at the outward curvature exit (0.40 ± 0.3 m/s), and nearly normal at the central isthmus (0.62 ± 0.2 m/s).

**Figure 1:**
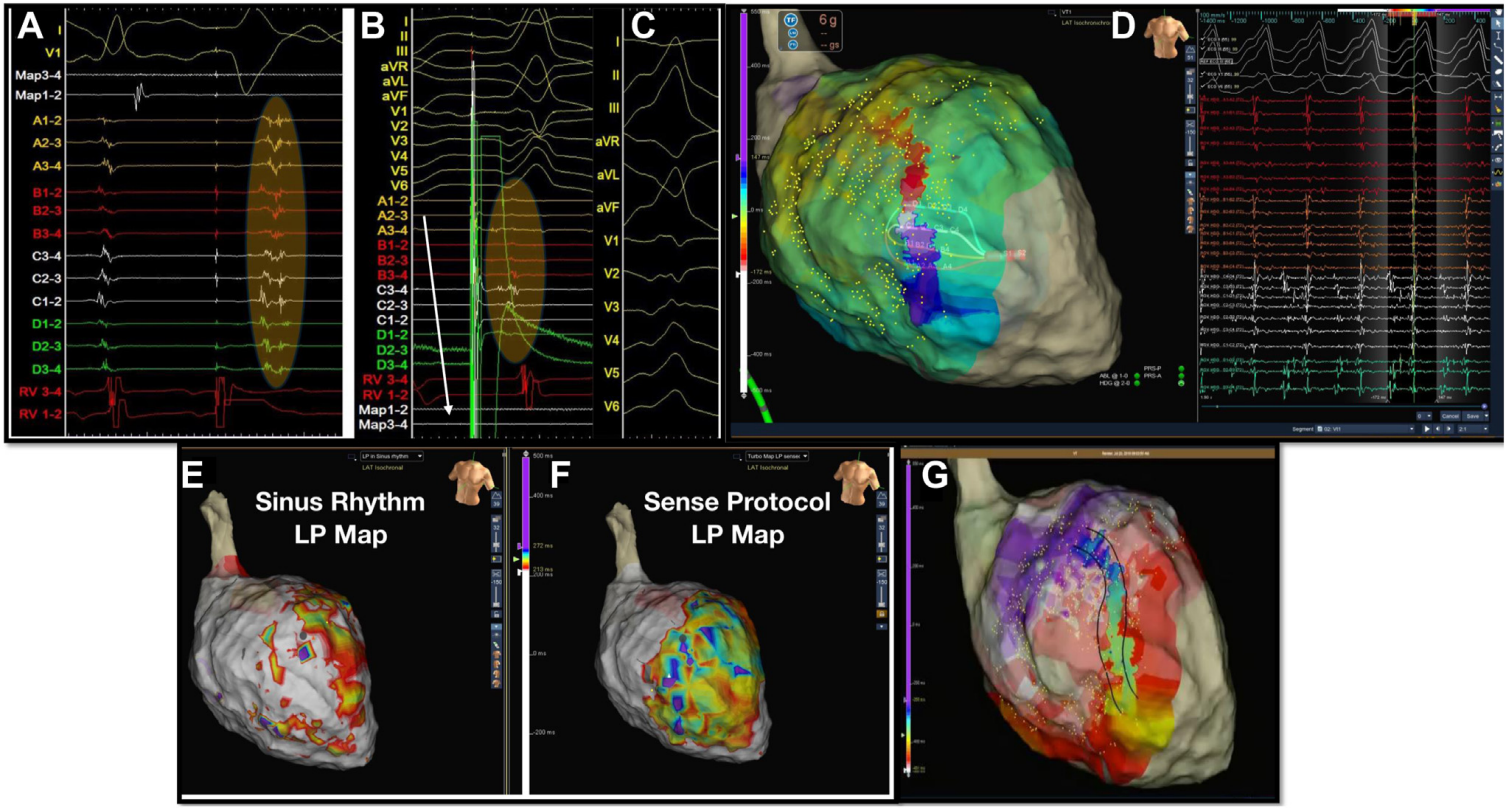
Functional behavior of late potentials (LPs) guided by the sense protocol (SP) in a patient with post–myocardial infarction ventricular tachycardia (VT). **A:** Functional substrate mapping delineating the hidden LPs. **B:** Pace mapping (12 of 12) from the exit site of the VT. **C:** Electrocardiography (ECG) of the VT. **D:** Activation mapping during VT using the HD Grid delineates mid-diastolic potentials identifying the isthmus of the re-entry circuit colocalized with the regions of hidden LPs guided by the SP. **E:** Activation mapping in sinus rhythm delineating LPs. **F:** Activation mapping using SP. **G:** Activation mapping during VT delineating the active VT circuit.

In the setting of post–myocardial infarction cardiomyopathy, specific EGM signatures are expressions of distinct electrophysiological phenomena. De Bakker and colleagues^37^ reported that these EGMs are the signature of small bundles of viable conducting tissue within a low-voltage area and that re-entry circuit isthmuses can be bordered by unexcitable scars that create conduction block. Frontera and colleagues^38^ elegantly demonstrated that LAVAs and LPs can belong to either the VT circuit or bystander areas, but that LPs with low amplitude and short duration appear linked to the VT isthmus (Figure 1). They concluded that LAVAs related to slow conduction areas are characterized by low-amplitude, fractionated, and long-duration EGMs, resulting from the low conduction velocities affecting the diseased area. However, once inside the protected isthmus, the EGM is represented by an LP of low amplitude and surprisingly short duration. Wavefront collision sites may also contribute to producing similar EGM signatures, with the resulting EGM the sum of the 2 opposing signals, with high-amplitude and short-duration fractionation.^38^

Martin and colleagues^23^ analyzed VT isthmuses in scar-related VT using high-density mapping, delineating that VT circuits have multiple entrances, exits, and dead ends. The local signal voltage was often higher in VT than sinus or paced rhythms. Thus, EGM characteristics are a challenge to interpret in intrinsic rhythm and yet may still play an important role in VT in which fiber recruitment is different.

## Conventional methods to identify critical regions

### Pace mapping to identify the critical VT isthmus

Pace mapping is a technique used to localize the exit site of postinfarct re-entry VT circuits.^39^^,^^40^ Brunckhorst and colleagues^39^ demonstrated that the pace-mapped QRS morphology from a catheter located at or near the exit of a VT re-entry circuit should usually produce a QRS morphology similar to that of VT. Pace mapping in sinus rhythm also provides a measure of slow conduction, indicated by the interval between the stimulus and the QRS complex (S-QRS) exceeding 40 ms. Sites with an S-QRS delay usually are in the infarct region, as identified by EGM voltage. It is likely that pacing sites with long S-QRS delays are in a potential isthmus, adjacent to regions of conduction block.^39^ Typically, the VT exit site pacing will yield a matched QRS morphology with a short S-QRS interval. As the pacing site moves from the exit region to the VT entrance site within the VT isthmus, the S-QRS interval theoretically gets longer.^39^^,^^40^

De Chillou and colleagues^41^ reported that an abrupt transition between a paced QRS complex that matches the clinical VT (exit site) and a nonmatched paced QRS complex (entrance site) identifies the core of the critical isthmus in scar-related VT (Figure 1). An abrupt change in QRS morphology is observed when the mapping catheter is moved from one side to the other of the mid-isthmus line. When pacing at the exit site of the mid-isthmus line, the activation wavefront propagates in both directions with respect to the isthmus orientation but more rapidly to the exit zone of the isthmus as compared with the entrance zone of the isthmus. Therefore, the myocardial depolarization outside the isthmus begins at the exit of the isthmus and spreads away from there to depolarize the ventricle, giving a QRS morphology resembling the VT.^41^^,^^42^

### Scar dechanneling

Characterization of scar regions is important for the initiation and maintenance of VT re-entry circuits, known as conducting channels (CCs).^43^, ^44^, ^45^ In a small series of patients with arrhythmogenic or ischemic cardiomyopathy, it has been suggested that radiofrequency ablation at the CC entrance using a scar dechanneling technique can target the scar without extensive ablation and potentially improve ablation efficiency. Berruezo and colleagues^46^ reported a new method for scar-related VT EGMs with delayed components, classified as entrance or inner conduction channel points, depending on delayed-component precocity during sinus rhythm. The CC entrance is defined as the EGM with delayed components with the shortest delay between the far-field component of healthy or SBZ muscle (low frequency, high voltage) and local component (delayed, high frequency, fractionated, and low voltage) corresponding to the local activation of myocardial fibers in the scar. They demonstrated that substrate ablation using the scar dechanneling technique resulted in low recurrence and mortality rates in more than half of patients.^46^

## Functionally guided substrate modification

### isochronal late activation mapping

Isochronal crowding is defined as local deceleration of propagation in a region within scar with bunching of isochrones (>2 isochrones within 1-cm radius).^47^ Within a given isochrone, the thinnest portion represents regions with the slowest conduction velocity. Therefore, localized isochronal crowding or “bunching” areas signify the greatest extent of fixed conduction slowing during sinus rhythm. Studies have shown that targeting earlier LPs frequently results in the elimination of delayed activation downstream in a channel.^44^^,^^48^ Recently, Irie and colleagues^47^ demonstrated that sites critical for re-entry in ventricular arrhythmias are frequently identified outside the latest isochrone of ventricular activation during sinus rhythm and are commonly localized to the surface or chamber mapped with the most delayed activation. The median percentage of the latest activation at critical sites was 78% at a distance from the latest isochrone of 18 mm. Sites critical to re-entry were harbored in regions with slow conduction velocity, in which 3 isochrones were present within a 1-cm radius.^47^ Isochronal late activation mapping were displayed with 8 equally distributed isochrones of activation (12.5% of ventricular activation comprised each isochrone).

#### Differentiating functional from anatomical block

As re-entrant VT requires fixed or functional localized conduction slowing, Brunckhorst and colleagues^49^ demonstrated with multiple wavefront mapping that regions with fixed and functional conduction delay may be equally critical for VT maintenance. The formation of functional block is dependent on heterogeneities of the electrophysiological properties of myocardial fibers.

**Figure 2:**
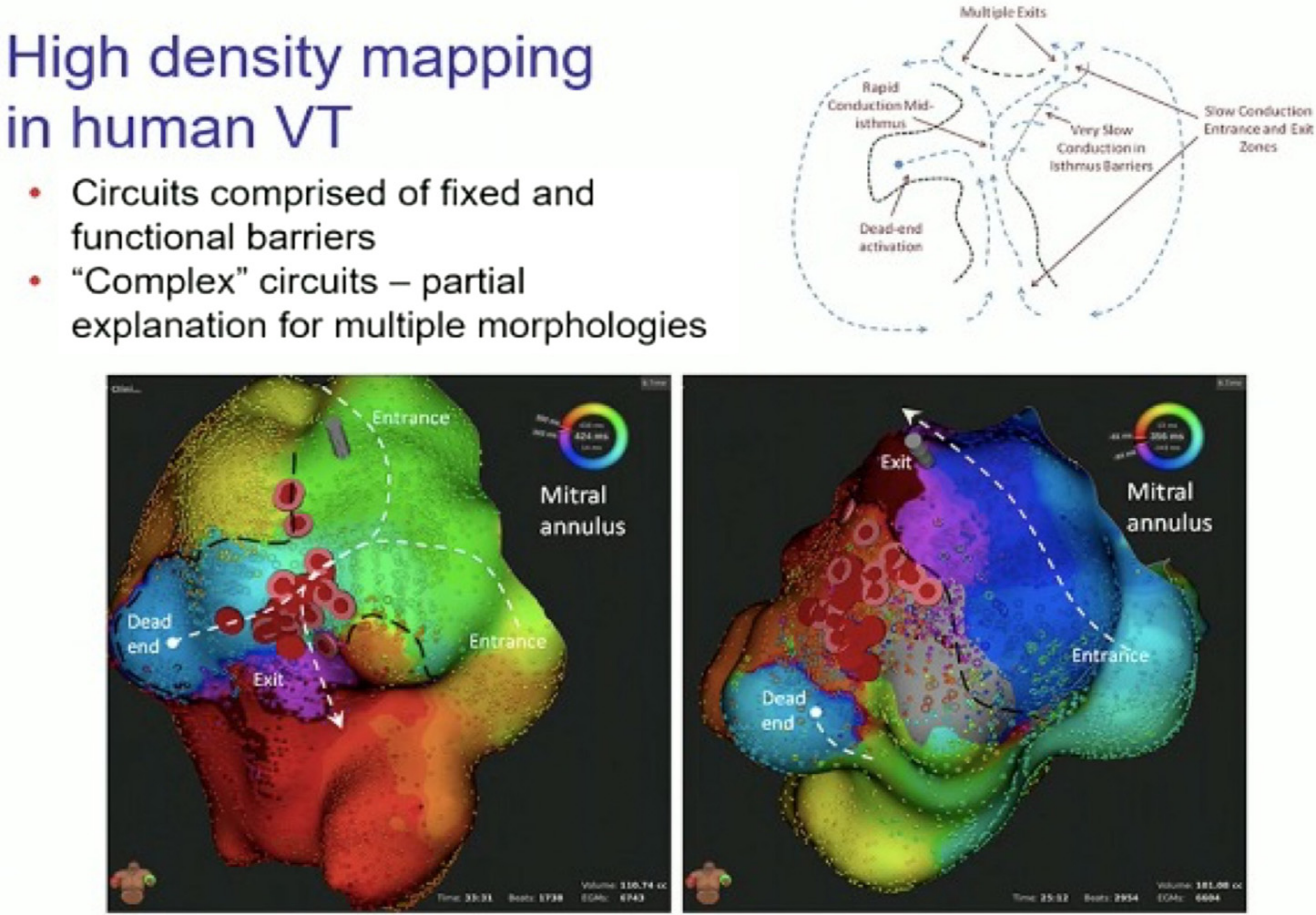
Change from Y-shaped to perimitral isthmus with ablation. **Left:** In the initial ventricular tachycardia (VT) (cycle length 424 ms), there is a Y-shaped isthmus with 2 entrances, a common longitudinal channel, and a single exit from one side of the channel. **Right:** After ablation (red dots), a faster (cycle length 356 ms) VT was induced, which passed from one entrance to the other of the original circuit as a circumferential perimitral VT.

Functional block has also been defined as an area of the myocardium that is not electrically excitable at shorter coupling intervals but is excitable at relatively long cycle lengths.^50^ There is often an overlap of lateral, functional block lines for 2 VT re-entry circuits with different electrocardiographic morphologies because the wavefront travels in opposite directions during the diastolic interval, alternating entrance and exit sites.^35^ There are often VT cycle-length variabilities and activation sequences between circuit morphologies due to changes in functional block related to the programmed stimulus location, the coupling interval between stimuli, and VT cycle length (Figure 2).^35^ Anatomical conduction block is present during sinus rhythm, pacing, and VT.^51^ These definitions, however, are not exact, as the anatomical block will not be apparent if the activation wave travels in parallel to it or intercepts at an oblique angle. Conversely, anatomical conduction block exists regardless of wavefront orientation and coupling interval.^35^

## Functional extrastimuli mapping

Among all identified LP in a given substrate, it is not known which zone of late activation most commonly provides the substrate for re-entry. In addition, LPs may be unrelated to any VT circuits and may represent unnecessary ablation targets. The most delayed LPs are not necessarily most functionally specific for re-entry.^47^ An elegant method for identifying sites vulnerable to conduction block and re-entry is mapping during premature ventricular stimulation. The principal idea of this method is to identify bundles with normal conduction properties during slow rates that are yet vulnerable to conduction block at faster rates (Table 2).

**Table 2:** Functional substrate mapping in VT ablation

Type of mapping	Etiology	Extrastimulus	Location of decremental properties	Ablation target	Acute clinical outcomes	Long-term follow-up
LAVA^16^	70 consecutive patients with ICM and DCM	1 extrastimulus VERP or 250 ms	LAVAs occupied 39 ± 32 cm^2^ of the 245 ± 174 cm^2^ of the LV surface (16%) Endocardial and epicardial LAVAs in 63 (90%) of 70 patients and 17 (81%) of 21 patients, respectively	LAVA elimination	Little agreement between noninducibility of VT and LAVA elimination as procedural end points (k statistic 0.038 ± 0.097; *P* = 0.70**)**	Median 22 mo: reduction in VT recurrence or death (hazard ratio 0.49; 95% confidence interval 0.26–0.95; *P* = .035)
ILAM^47^	33 patients with ICM, nonischemic cardiomyopathy, and ARVD, 10 consecutive patients 10 consecutive patients ILAM-guided VT ablation	N/A	N/A	ILAM (10 patients**)**	Complete noninducibility in 40%, elimination of the clinical VT in 50%	6 ± 1 mo: 80% of the patients free of VT recurrence (50% free of antiarrhythmics
DEEP^59^	20 patients with ICM	1 extrastimulus, VERP +20 ms	In 50% of DEEP areas colocalized with the diastolic isthmus	Only DEEP	High noninducibility (80%)	6 mo: mean VT burden reduced to 0 from 11 preprocedure
HSC^61^	37 patients with ICM, ARVD, DCM or scar in MRI	2–3 extrastimuli, VERP +60 ms VERP +40–20 ms, and VERP +10–20 ms	Majority (53%) in border zone areas, 18.2% within dense scar regions, 28.8% in normal-voltage tissue surrounding the scar area	Both LPs and HSC, and compared with historical cohort of LP only	Low inducibility (24.3% vs 48.7%)	6 mo: VT-free survival: 13.5% vs 20%
RV SP single extra pacing^60^	30 patients with ICM	Single sensed extrastimuli +20 ms VERP every fifth beat	LP 9% of the total scar in SR and 38% during SP	Ablation to sites of best entrainment/pace map and all LP and LAVA substrates defined by the Barts SP	29 (97%) of 30 patients noninducibile	12 mo: 90% free from ATP or ICD shocks
HSC^58^	60 patients with ICM	One extrastimuli, VERP +50 ms	37% of EDPs in areas with normal BV, 91 within the scar and 71% in a region of nontransmural scar based on MRI	Only EDP elimination	67% noninducibility, 23% induced VTs were successfully abolished based on activation, entrainment, or pace mapping, 10% inducibility	16 mo: low recurrence (22%)

ATP = antitachycardia pacing; ARVD = arrhythmogenic right ventricular dysplasia; BV = bipolar voltage; DCM = dilated cardiomyopathy; DEEP = decrement evoked potential; EDP = evoked delayed potentials; HSC = hidden slow conduction; ICD = implantable cardioverter-defibrillator; ICM = ischemic cardiomyopathy; ILAM = isochronal late activation mapping; LAVA = local abnormal ventricular activity; LP = late potential; LV = left ventricular; MRI = magnetic resonance imaging; N/A = not available; RV = right ventricular; SP = sense protocol; VERP = ventricular effective refractory period; VT = ventricular tachycardia.

The insight that decrement precedes unidirectional block was first described in atrial tissue sections in studies undertaken by Lammers and colleagues.^52^ Studies showing the same results were also demonstrated in experimental models and humans.^53^, ^54^, ^55^ Recently, Haïssaguerre and colleagues^56^ demonstrated that extra stimulation in patients with Brugada syndrome caused the disappearance of epicardial EGMs at multiple localized sites. This was not observed in endocardial right ventricular regions where EGMs prolonged at short coupling intervals but remained present.^56^

Jackson and colleagues^57^ reported that substrate mapping using extrastimulation is an effective strategy for interrogating the substrate and localizing sites that form functional block during VT. Furthermore, mapping strategies incorporating ventricular extrastimulation have been shown to increase the accuracy of substrate mapping for identifying VT isthmus sites.^16^^,^^58^, ^59^, ^60^ The technique has been refined by groups, using it both to unmask hidden substrate in patients with small or nontransmural scars^16^ or to allow targeting of LAVAs presumed responsible for VT circuits within large scars.^59^

Acosta and colleagues^61^ demonstrated that the evoked response to a double ventricular extrastimulus during substrate mapping allowed the identification of areas of myocardium having slow and decremental conduction properties that are hidden during sinus rhythm. The regions with hidden slow conduction EGMs (HSC-EGMs) were found not only in SBZs, but also in areas labeled as normal-voltage tissue. In signal intensity maps obtained from contrast-enhanced cardiac magnetic resonance, HSC-EGMs were located within scar regions, and scar dechannelling incorporating HSC identification and ablation was associated with a shorter radiofrequency time and a lower VT inducibility rate after substrate ablation.^61^

**Figure 3:**
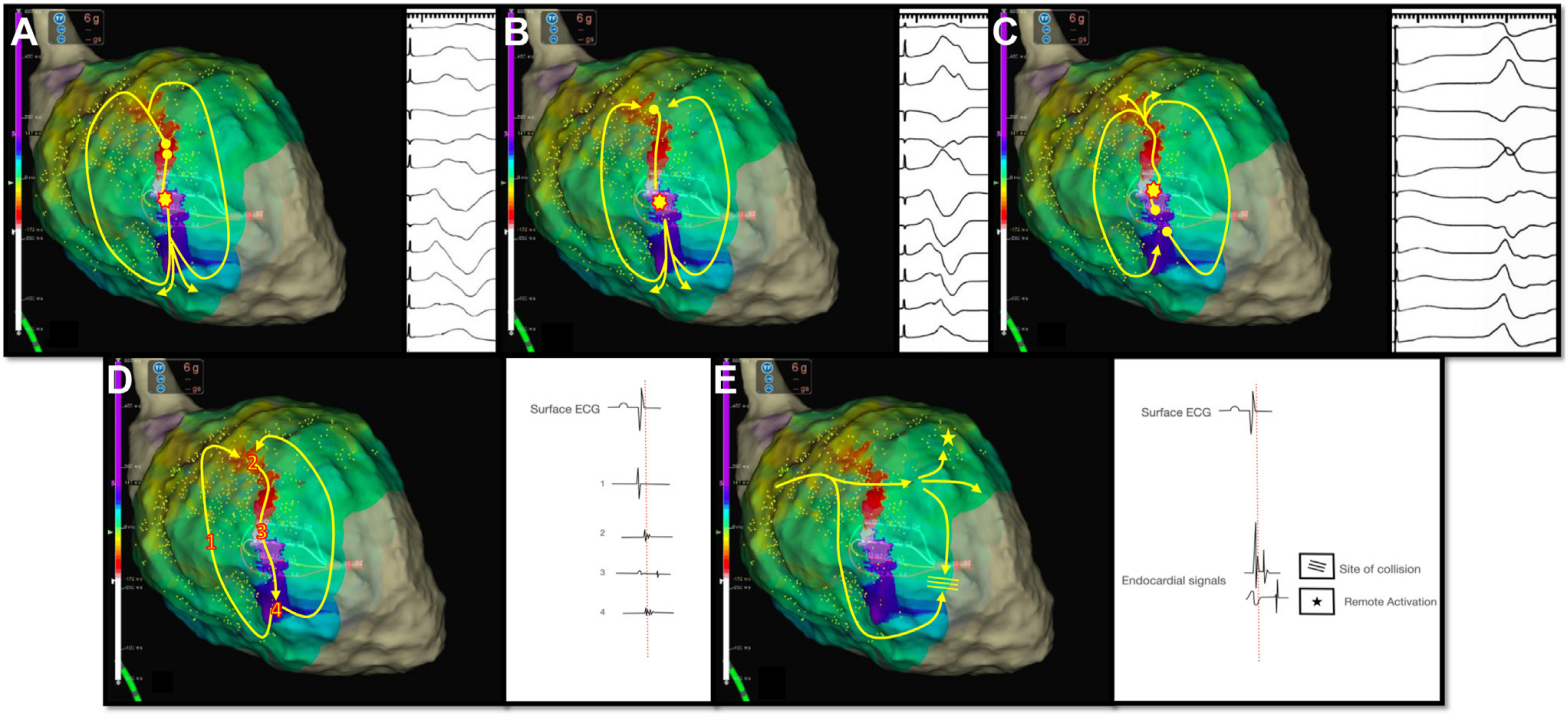
The value of pace mapping and electrogram (EGM) morphology to identify the active ventricular tachycardia (VT) circuit. **A:** Pacing from the critical isthmus produces a wavefront propagating only to the exit site of the active VT circuit, giving a perfect match with our clinical VT. **B:** Pacing from the same site with the same drive train and a premature ventricular extrastimulus the wavefront propagates simultaneously to the entrance and exit site, giving a variably fused morphology compared with the QRS morphology of the clinical VT. **C:** Pacing from the same site with a different coupling interval of the premature ventricular extrastimulus, the wavefront propagates only from the entrance site of the active VT circuit, producing a different QRS morphology. **D:** EGM morphologies at the entrance (2), mid-isthmus (3), exit site (4), and the outer loop (1) of the active circuit during high density mapping with HD Grid in sinus rhythm. **E:** EGM morphologies in a collision site and a site of remote bystander activation not related with the active circuit during high-density mapping with HD Grid in sinus rhythm. LP = late potential.

Functionally guided substrate modification approaches that consider both activation and voltage patterns guided by extrastimuli (sense protocol) have been described as alternatives to extensive ablation required to homogenize scar (Figure 3, Video 1).^60^ Srinivasan and colleagues^60^ created 2 substrate maps, 1 during sinus rhythm and 1 mapping the paced beat of a single sensed extra from the right ventricular apex to invoke left ventricular conduction delay. Ablation rendered VT non-inducible in 29 of 30 patients, and 90% of patients were free from VT. Figure 3 shows an example of functional substrate mapping in a patient with infarct-related VT, in which LPs and LAVAs observed using the SP were able to identify regions critical for ablation in VT with greater accuracy than mapping in sinus rhythm.

**Figure 4:**
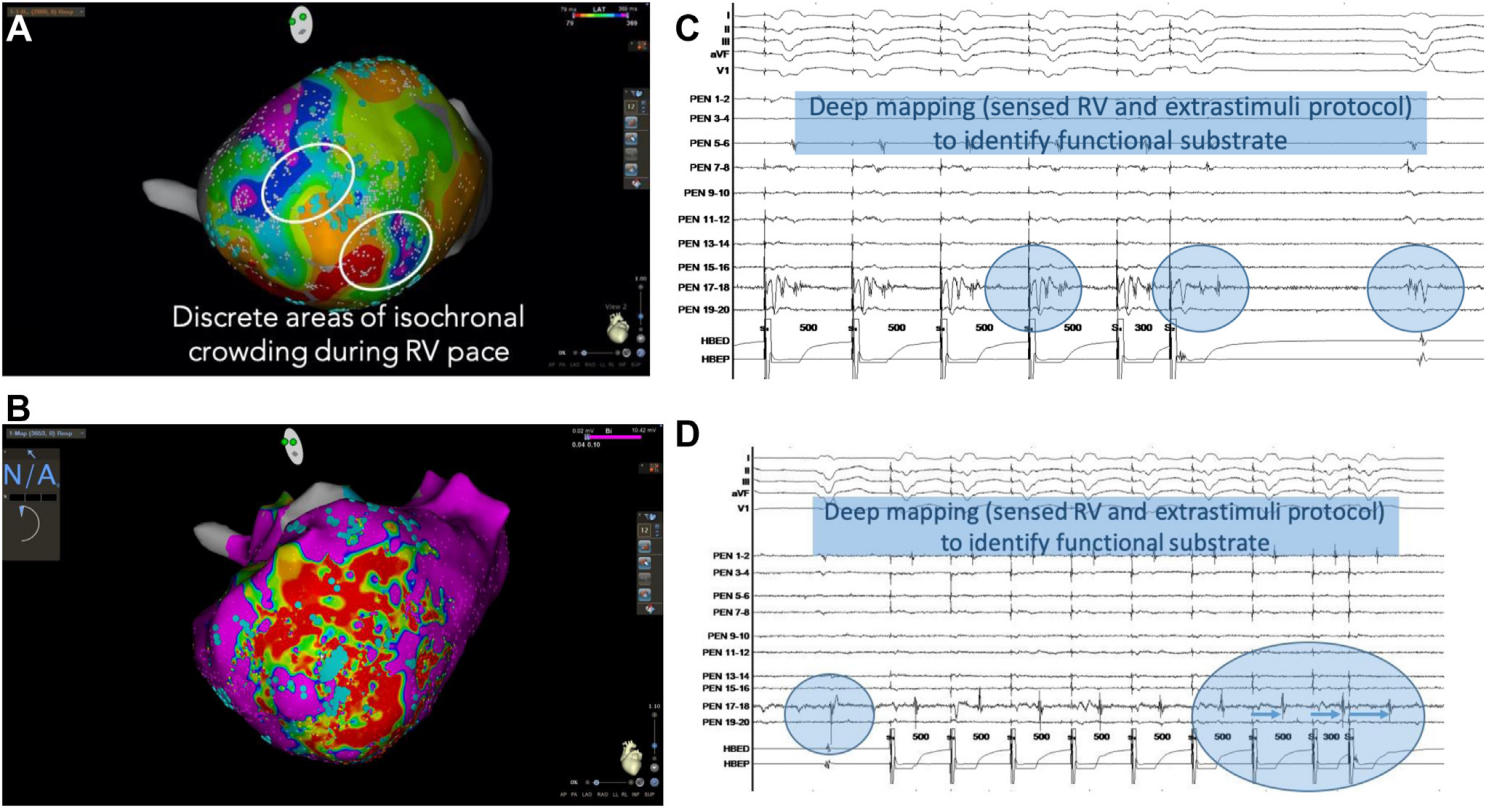
Functional substrate mapping in a patient with infract-related ventricular tachycardia (VT). **A:** Discrete regions of isochronal crowding during right ventricular (RV) pacing (cycle length 500 ms) in the left ventricular apex. Sites critical to re-entry were harbored in regions with slow conduction velocity, in which 3 isochrones were present within a 1 cm radius. **B:** Voltage mapping in the same patient, scar tissue <0.04 mV, low-voltage regions 0.04–0.1 mV, border zones >0.1 mV. **C, D:** Decrement evoked potential (DEEP) mapping using a sensed RV and extrastimuli protocol allowed us to identify the functional substrate perpetuating VT re-entry.

Porta-Sánchez and colleagues,^59^ in a multicenter prospective study, identified the functional substrate critical to the VT circuit using decrement evoked potential (DEEP) mapping with extrastimulus substrate mapping. This technique using DEEP substrate mapping identified the functional substrate critical to the VT circuit with high specificity (Figure 4). They found that areas of DEEPs are localized more frequently in the diastolic pathway of the VT than LPs and that targeting the DEEP regions deemed VT noninducible in the majority of cases. Midterm outcomes were in keeping with the current ablation outcomes described in the published data.

## Effect of activation wavefront on EGM characteristics

EGM annotation methods based on the maximal negative derivative of the extracellular potential or maximal voltage may be inaccurate in nonuniform anisotropic tissue. Detected EGMs are strictly dependent on electrode orientation, respective to wavefront propagation, which rapidly changes directions resulting in a changeable recording according to the vector line of assessment. As a result, catheters may not identify low-voltage EGMs propagating orthogonal to their orientation, potentially missing critical arrhythmic substrate.

Arenal and colleagues^6^ were the first to show vector-dependent recording of isolated and late ventricular activities showing that the substrate of scar-related VT is linked to interconnected activation propagating from the SBZ with increasing delay. Tung and colleagues^62^ have recently investigated the impact of the activation wavefront direction on the values obtained during voltage mapping. They observed significant differences in the bipolar and unipolar low-voltage characterization of a scar with different ventricular activation wavefronts, particularly in septal locations and in patients with heterogeneous as opposed to dense scar. An alternate activation wavefront increased the sensitivity to detect arrhythmogenic substrate and critical areas for VT. One limitation of this study was the use mainly of 3.5-mm irrigated-tip ablation catheters for mapping, which increases far-field recording and reduces accuracy in comparison with using multipolar catheters with short interspaced electrode.^26^^,^^63^

In the study by Martin and colleagues,^23^ the diastolic signal voltage in the mid-isthmus was lower in sinus or paced rhythms than in VT. This difference may be explained by changes in the direction of wavefront propagation with respect to the recording electrodes; most isthmuses seem to be orientated longitudinally in the ventricle, and recordings are made between electrode pairs on the same spline, which is longitudinally oriented on the Orion multipolar catheter, and so wavefronts during VT may be more likely to be orientated perpendicularly to the recording electrode pair. Alternatively, there may be a true difference in signal amplitude in the mid-isthmus during VT because of differences in fiber recruitment between rhythms. During VT, activation in scar is mostly in one direction, often aligned with the endocardial muscle fiber orientation: this reduction in wavefront collision may improve fiber recruitment. Further, shortened fiber refractory periods during tachycardia and protection of the mid-isthmus by lines of functional block may allow recruitment of more fibers during tachycardia than in sinus or paced rhythms, increasing local signal voltage.^23^

Brunckhorst and colleagues reported that changing the activation sequence (from atrial pacing to ventricular pacing) produced a >50% change in EGM amplitude at 28% of sites and a >100% change at 10% of sites, but only 8% of sites had an EGM amplitude classified as abnormal (≤1.5 mV) with one activation sequence and normal (>1.5 mV) with the other activation sequence. Electrically unexcitable scar (6% of sites) was associated with lower EGM amplitude but could not be reliably identified based on EGM amplitude alone for either activation sequence.^64^ We recently demonstrated that in post–myocardial infarction VT, wavefronts of conduction slowing or block might aid the identification of critical isthmuses to target ablation therapy in unmappable VTs.^24^ Lines of block corresponding to the SBZ can be more easily visualized with pacing resulting in a wavefront of activation perpendicular instead of parallel to the VT isthmus. The region of the latest activation in a paced map colocates with the location of the VT isthmus independently from the pacing site.

### Clinical implications of new technologies: Future perspectives

SURVIVE-VT (Substrate Ablation versus Antiarrhythmic Drug Therapy for Symptomatic Ventricular Tachycardia) has shown that a substrate-based catheter ablation procedure was associated with a significantly lower rate of implantable cardioverter-defibrillator shocks, cardiovascular death, and hospitalization for worsening heart failure.^65^ Current international guidelines do not provide clear recommendations for preventing VT recurrences.^66^^,^^67^ Recently, Tung and colleagues^68^ demonstrated that early catheter ablation performed at the time of implantable cardioverter-defibrillator implantation significantly reduced the composite primary outcome of VT recurrence, cardiovascular hospitalization, or death enrolling patients with cardiomyopathy of varied etiologies.

It remains to be seen whether functional substrate mapping may further improve patient outcomes in an early ablation strategy, but as a technique, it would be optimally placed to be used as a first-line safe strategy for substrate guided VT ablation.

**Figure 5:**
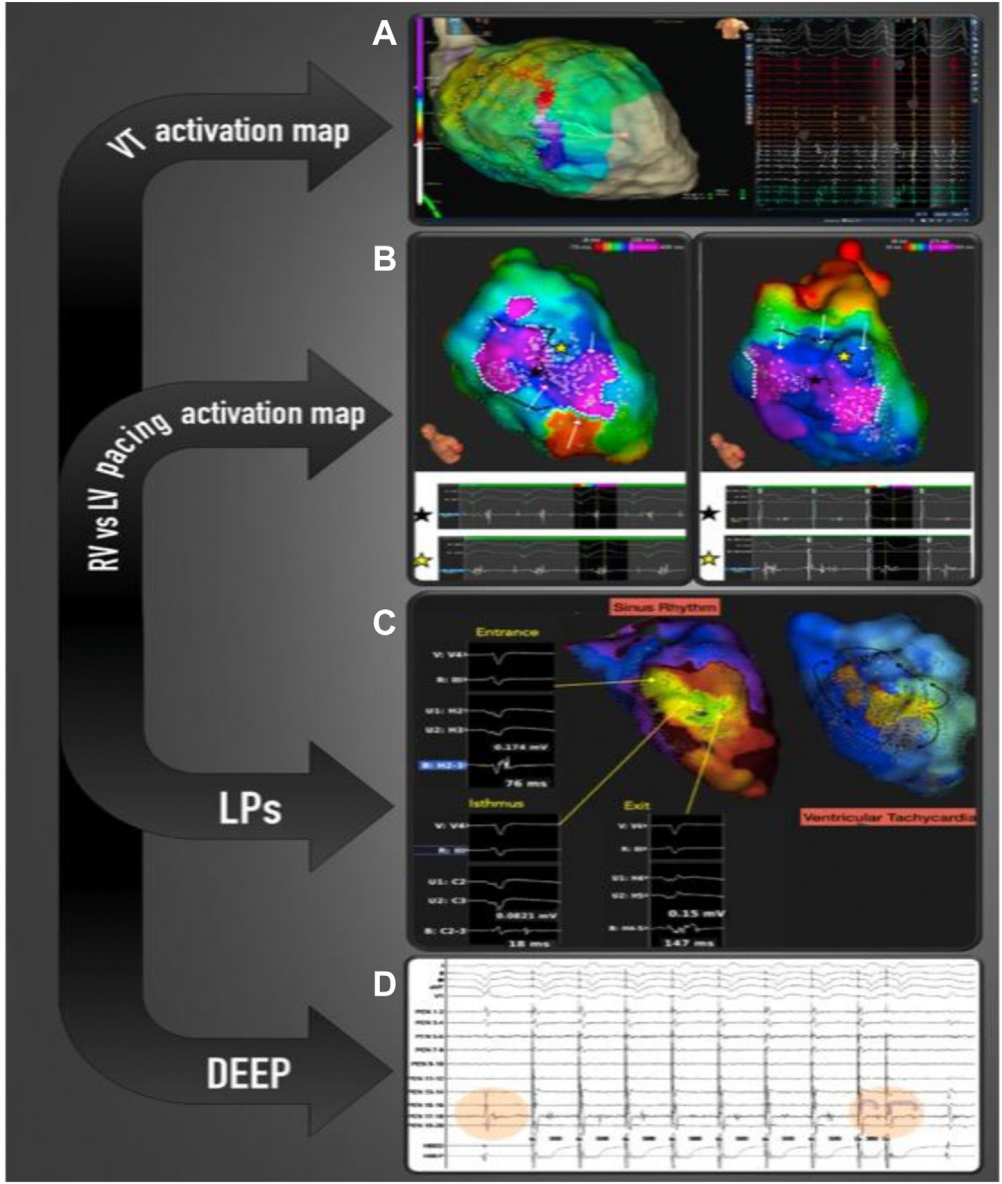
Different mapping techniques used to identify potential re-entry ventricular tachycardia (VT) circuits critical for the initiation and maintenance of the ventricular arrhythmia. **A:** Activation mapping of the VT. **B, C:** Activation mapping during right ventricular (RV) and left ventricular (LV) pacing delineating the change of direction of conduction using different pacing sites and identifying local abnormal ventricular activity (LAVA) and late potential (LP). **D:** Decrement evoked potential (DEEP) mapping identifying potential the critical isthmus of the VT.

Additionally advances in mapping technology continue to play a key role in improving resolution of functional signals. Multipolar electrode catheters with 1-mm electrodes and variable interspacing have been introduced to overcome limitations in mapping in a unidirectional manner. The Advisor HD Grid Mapping Catheter (Catheter Sensor Enabled) is a multipolar catheter with electrodes equally interspaced along each spline and arranged in a grid configuration. The catheter simultaneously records EGMs along and across different orthogonal vectors when point acquisition is set in the bidirectional WAVE configuration. As well as enabling an increase in the near-field-to-far-field signal ratio, it can provide omnipolar EGMs that are independent of the incident wavefront and the fixed interelectrode spacing along and across splines, allowing calculation of conduction velocities.^69^ The Optrell catheter (Biosense Webster) which shortly will be released, has similar properties. The use of these catheters in combination with novel mapping algorithms, allowing automated sequential pacing from each electrode at a time and recording from all the remainder electrodes, can rapidly identify sites vulnerable for conduction block. This method can be repeated at shorter coupling intervals, providing a rapid method for evaluating the influence of wavefront direction and coupling interval on conduction properties. The advantages of multipolar mapping catheters with multiple small electrodes and narrow interelectrode distance are (1) higher mapping density and better substrate definition, (2) higher detection of LAVAs and voltage channels, and (3) higher accuracy in identifying and delineating near-field component (LAVAs) and differentiating from far-field signals.^26^ In addition, the multipolar mapping catheters have been coupled with dedicated algorithms that automatically highlight areas with EGMs having specific characteristics or timings (eg, Lumipoint and RHYTHMIA; Boston Scientific). These features may enhance human interpretation of the EGM signals during a case, particularly in which the circuit lies in partial scar with low-amplitude near-field signals, and potentially allow a more targeted ablation strategy. Functionally guided substrate modification approaches that consider both activation and voltage patterns guided by extrastimuli (sense protocol) have been described as alternatives to extensive ablation required to homogenize scar (Figure 3, Video 1). Porta-Sánchez and colleagues,^59^ in a multicenter prospective study, found that areas of DEEPs are localized more frequently in the diastolic pathway of the VT than LPs and that targeting the DEEP regions deemed VT noninducible in the majority of cases (Figure 4). These findings support the need for prospective randomized studies to compare conventional activation mapping during VT and substrate mapping targeting LAVAs or LPs with novel mapping techniques using DEEP mapping or sense protocol pacing from different pacing sites using novel multipolar catheters to inform the development of our mapping and ablation strategies for VT (Figure 5).

## Conclusion

Current substrate modification procedures employing extensive substrate modification covering the entirety of the abnormal substrate are associated with long procedural times and may be unfeasible in unstable patients with limited cardiac reserve and large substrates.^70^ We need further data and randomized studies to understand the value of focused DEEP or sense protocol mapping along with automated annotation, which may be associated with a more targeted ablation strategy and potentially improve ablation outcomes.
